# Serum Anion Gap Is Associated with All-Cause Mortality among Critically Ill Patients with Congestive Heart Failure

**DOI:** 10.1155/2020/8833637

**Published:** 2020-11-16

**Authors:** Yiyang Tang, Wenchao Lin, Lihuang Zha, Xiaofang Zeng, Xiaoman Zeng, Guojun Li, Zhenghui Liu, Zaixin Yu

**Affiliations:** ^1^Department of Cardiology, Xiangya Hospital, Central South University, Changsha, Hunan, China; ^2^Department of Cardiology, Wenchang People's Hospital, Wenchang, Hainan, China; ^3^Department of Emergency, The Third Hospital of Changsha, Changsha, Hunan, China; ^4^Department of Neurology, Xiangya Hospital, Central South University, Changsha, Hunan, China; ^5^National Clinical Research Center for Geriatric Disorders (Xiangya), Changsha, Hunan, China

## Abstract

**Background:**

Congestive heart failure (CHF) is a complex clinical syndrome, with high morbidity and mortality. Serum anion gap (SAG) is associated with the severity of various cardiovascular diseases. However, the role of SAG indicators in CHF is unclear.

**Methods and Results:**

A retrospective analysis of data from Multiparameter Intelligent Monitoring in Intensive Care III version 1.4 was conducted in critically ill patients with CHF. The clinical information of each patient, including demographic data, comorbidities, vital signs, scores, and laboratory indicators, were successfully obtained. Cox proportional hazards models were used to determine the relationship between SAG and mortality in patients with CHF, the consistency of which was further verified by subgroup analysis.

**Results:**

A total of 7426 subjects met the inclusion criteria. Multivariate analysis showed that after adjusting for age, gender, ethnicity, and other potential confounders, increased SAG was significantly related to an increase in 30- and 90-day all-cause mortalities of critically ill patients with CHF compared with decreased SAG (tertile 3 versus tertile 1: adjusted hazard ratio, 95% confidence interval: 1.74, 1.46–2.08; 1.53, 1.32–1.77). Subgroup analysis indicated that the association between SAG and all-cause mortality presented similarities in most strata.

**Conclusion:**

SAG at admission could be a promising predictor of all-cause mortality in critically ill patients with CHF.

## 1. Introduction

Congestive heart failure (CHF) is the end-stage manifestation of various cardiovascular diseases with structural and functional disruptions in the myocardium, causing restricted ventricular ejection or filling, systemic blood circulation disorder, insufficient perfusion of tissues and organs, and ultimate death [1]. CHF has become a major public health problem with more than 23 million patients affected worldwide, and it still has a high mortality despite great progress in diagnosis and treatment for the last few decades [2]. Thus, how to early identify high-risk patients, determine their prognosis, and formulate effective individualized interventions have attracted growing attention from clinicians in recent years.

Metabolic acidosis is a common complication of CHF; it is closely related to ischemia and hypoxia of tissue caused by hemodynamic disorders and the use of diuretics [3]. In turn, acidosis and accompanying hyperkalemia could further weaken myocardial contractility, creating a vicious circle. Acidosis serves as the independent predictor for the long-term prognosis of CHF, and pH value could help to stratify the risks [4]. However, the predictive value of other laboratory parameters that reflect the acid-base imbalance in CHF requires more evidence, such as serum anion gap (SAG).

SAG refers to the difference between undetermined anions and cations. It indicates the concentration of fixed acids in plasma, and it is a commonly used and easily obtained laboratory parameter of acid-base imbalance [5]. Some recent studies have confirmed that SAG is elevated and closely associated with poor prognosis of various diseases, including acute pesticide poisoning [6], sepsis [7], acute and chronic kidney injury [8, 9], trauma [10], and coronary artery disease [11]. However, whether the anion gap could be used as a prognostic marker for critically ill patients with CHF remains unclear. Therefore, the present study is aimed at investigating the association between SAG and mortality in these patients.

## 2. Materials and Methods

### 2.1. Data Source

The Multiparameter Intelligent Monitoring in Intensive Care III version 1.4 (MIMIC-III v1.4) is a freely accessible critical care database developed and operated by the Massachusetts Institute of Technology; it contains detailed clinical data of 53,423 adult patients (age more than 16 years) from June 2001 to October 2012 in the intensive care units (ICUs) of Beth Israel Deaconess Medical Center [12]. Before implementing the present research, author Tang completed and passed the CITI “Data or Specimens Only Research” course (No. 9014457) and obtained authorization for database access. In need of special note here is that this database was approved by the Institutional Review Boards of Massachusetts Institute of Technology (Cambridge, MA, USA) and Beth Israel Deaconess Medical Center (Boston, MA, USA), and no additional ethical approval needed to be provided.

### 2.2. Study Population and Design

**Figure 1:**
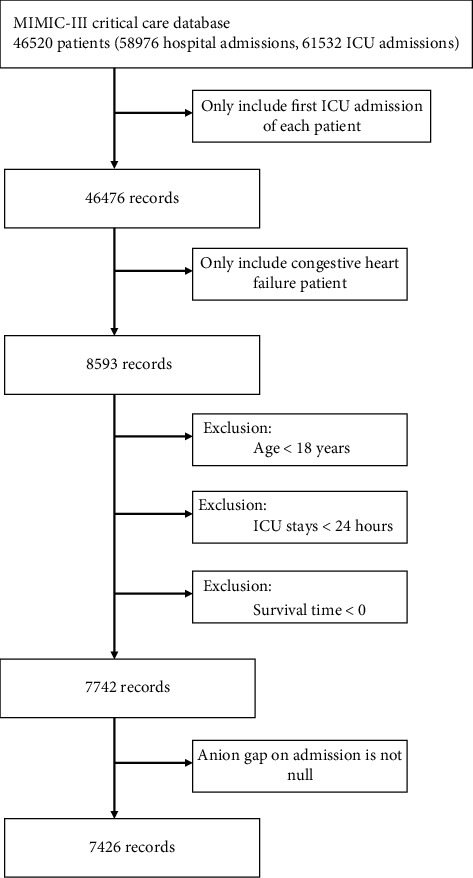
Workflow of the data extraction. The inclusion and exclusion criteria of the study subjects were revealed, and 7426 subjects were finally included. ICU: intensive care unit.

In the MIMIC-III database, all adult patients aged over 18 years old, first admitted to the ICU, and diagnosed with CHF were included in the study in accordance with the ninth revision of the International Classification of Diseases (ICD) code (code = 428.0). Patients with any of the following criteria were excluded from the study: (1) ICU stay time of less than 24 hours, (2) no anion gap results within 24 hours of admission to the ICU, and (3) survival time of less than 0 (some organ donors may die earlier than admission). The workflow is shown in Figure 1.

The starting point of the study was defined as the time of admission to the ICU, and the endpoint was the time of death, 30, or 90 days after admission. The patients' 30- and 90-day mortalities were chosen as the primary outcome of interest, and death data were extracted from the Social Security Death Index. The secondary outcomes were the readmission rate and the composite outcome of major adverse cardiac events (MACEs), including all-cause mortality, hospitalization with acute heart failure, heart transplantation, and mechanical circulatory support [13].

### 2.3. Data Extraction and Preparation

Structured query language (SQL) was utilized to extract clinical data with PostgreSQL tools (version 9.6), including demographics, comorbidities, vital signs, severity scores, laboratory tests, and interventions. Demographics included age, gender, and ethnicity. For the privacy protection of patients, the database has had the date of birth shifted to exactly 300 years for patients older than 89 years. Before the analysis, the age of these patients was adjusted in accordance with the following formula: realage = age − 300 + 89 [14]. Vital signs consisted of temperature, respiratory rate (RR), heart rate (HR), systolic blood pressure (SBP), diastolic blood pressure (DBP), mean blood pressure (MBP), and percutaneous oxygen saturation (SpO_2_). Comorbidities included acute myocardial infarction (AMI), atrial fibrillation, valvular heart diseases, pulmonary circulation diseases, hypertension (HBP), diabetes, pneumonia, respiratory failure, liver disease, renal failure, stroke, and malignancy. The sequential organ failure assessment (SOFA) score and the simplified acute physiology score II (SAPSII) were calculated for each patient when entering the ICU to assess the severity of the disease [15, 16]. Laboratory tests within 24 hours after ICU admission were also extracted, including SAG, white blood cell (WBC), platelet, hemoglobin, blood urea nitrogen (BUN), creatinine, sodium, potassium, chloride, bicarbonate, glucose, prothrombin time (PT), activated partial thromboplastin time (APTT), lactate, and plasma N-terminal probrain natriuretic peptide (NT-proBNP). Interventions included vasopressor, dialysis, and mechanical ventilation.

After extraction was performed, the raw data were merged and disposed with patient identifiers by using STATA version 16 (https://www.stata.com/). The “winsorize” function was used to reduce the effect of outliers, and multivariate multiple imputation with chained equations was utilized to impute the missing values. The details of the missing value are shown in Table 1. More than 20% of the subjects in this study cohort did not have records of lactate and NT-proBNP, which was converted and considered as a dummy variable in the models to avoid possible bias caused by direct filling of missing values [17].

### 2.4. Statistical Analysis

The baseline data of all subjects were divided into three groups by SAG tertiles. Continuous variables were presented as mean ± SD or medians and interquartile range. Kruskal-Wallis test was used to conduct a hypothesis test for continuous variables. Categorical variables were expressed by numbers and percentages, which were analyzed using Chi-square (or Fisher's exact) tests. A generalized additive model (GAM) was used to determine the nonlinear association between SAG and 30-day all-cause mortalities in critically ill patients with CHF. In addition, the relationship between SAG and the survival in patients with HF was visually shown through the Kaplan–Meier (K–M) curve and tested using the log-rank test.

Cox proportional hazard model was used to analyze the relationship between SAG and 30-day and 90-day all-cause mortality in critically ill patients with HF and determine the independent prognostic value of SAG in these patients, with the first tertile or quartile as the reference and adjusting for potential confounders. Two multivariate models were conducted, and the results were described as hazard ratio (HR) with 95% confidence intervals (CIs). In model I, the covariates were only adjusted for the confounders' age, sex, and ethnicity. On the basis of model I, model II was further adjusted for other confounders, including temperature, HR, RR, SBP, DBP, SpO_2_, weight, atrial fibrillation, liver disease, valvular heart diseases, pulmonary circulation diseases, pneumonia, respiratory failure, diabetes, stroke, malignancy, SOFA, SAPSII, lactate, NT-proBNP, PT, WBC, BUN, creatinine, potassium, bicarbonate, glucose, vasopressor, dialysis, and mechanical ventilation. These factors were chosen as confounders on the basis of their association with the outcomes or a change in effect estimate exceeding 10% [18]. Variance inflation factor was used to test the collinearity between variables with 5 as the threshold, and the variable MBP and chloride were deleted. The associations between SAG and readmission rate and MACEs were determined using multivariate logistic regression with results expressed as the odds ratio (OR) with 95% CIs. Besides, a subgroup analysis of the correlation between SAG and 30-day all-cause mortality was performed to examine whether the effect of SAG in various subgroups differed. All statistical analyses were performed on EmpowerStats version 2.20 (http://www.empowerstats.com/cn/, X&Y solutions, Inc., Boston, MA) and R software version 3.4.3; *P* < 0.05 (two-sided) was considered statistically significant.

## 3. Results

### 3.1. Clinical Characteristics of Subjects

A total of 7426 subjects were included in this retrospective study, 3971 of whom were male and 3455 were female. The age of the subjects was generally high, with a median of 75.3 years old. The subjects were mostly white, accounting for 73.3 percent share of the total. And the 30- and 90-day overall mortalities were 17.7% and 24.4%, respectively. The clinical characteristics of these subjects stratified by SAG tertiles are shown in Table 2. A total of 2334 subjects were in the low-SAG group (tertile 1, SAG < 13), 2519 subjects were in the mid-SAG group (tertile 2, SAG ≥ 13, and SAG < 16), and 2573 subjects were in the high-SAG group (tertile 3, SAG ≥ 16). Subjects with higher SAG levels had more comorbidities of HBP, AMI, diabetes, renal failure, and respiratory failure, with higher mortality, higher SOFA and SAPSII scores, and higher rates of use of vasopressor, dialysis, and mechanical ventilation.

### 3.2. Primary Outcome: Association between SAG and All-Cause Mortality

**Figure 2:**
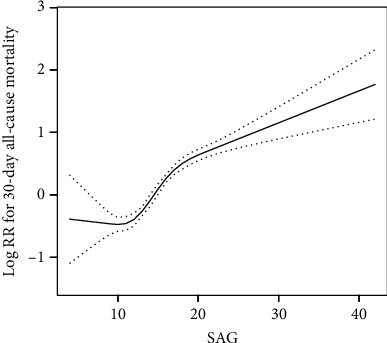
Construction of the smooth curve fitting of the risk of 30-day all-cause mortality and SAG using a generalized additive model. Dashed curves were for the 95% of confidence interval.

**Figure 3:**
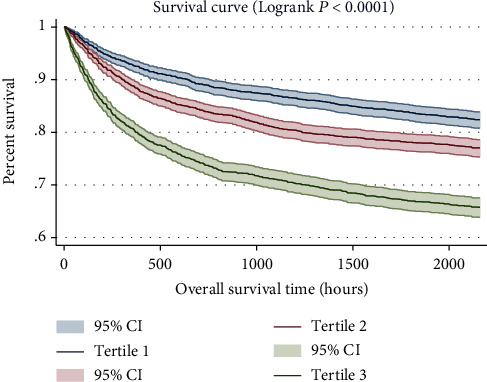
Kaplan–Meier survival curves for critically ill patients with CHF based on tertile of SAG. *x*-axis: survival time (hours). *y*-axis: cumulative survival probability. CHF: congestive heart failure. SAG: serum anion gap.

As shown in Figure 2, the result of GAM analysis indicated a U-shaped relationship between SAG and 30-day all-cause mortality in critically ill patients with CHF. The K–M survival curve illustrated that subjects with increased SAG levels presented a decreased survival rate and shortened survival time (Log-rank *P* < 0.0001, Figure 3).

The Cox proportional hazard model was used to assess the association between SAG and all-cause mortality (Table 3). In model I, after adjusting for age, sex, and ethnicity, high levels of SAG were significantly associated with increased risk of 30- and 90-day all-cause mortalities (tertile 3 versus tertile 1: HR, 95% CI: 2.62, 2.27–3.03; 2.23, 1.98–2.51; quartiles 4 versus quartiles 1: HR, 95% CI: 3.20, 2.68–3.82; 2.62, 2.27–3.02). In model II, after adjusting for age, sex, ethnicity, temperature, HR, RR, SBP, DBP, SpO_2_, weight, atrial fibrillation, liver disease, valvular heart diseases, pulmonary circulation diseases, pneumonia, respiratory failure, diabetes, stroke, malignancy, SOFA, SAPSII, lactate, NT-proBNP, PT, WBC, BUN, creatinine, potassium, bicarbonate, glucose, vasopressor, dialysis, and mechanical ventilation, high levels of SAG were still an independent predictor of 30- and 90-day all-cause mortalities (tertile 3 versus tertile 1: HR, 95% CI: 1.74, 1.46–2.08; 1.53, 1.32–1.77; quartiles 4 versus quartiles 1: HR, 95% CI: 1.97, 1.59–2.45; 1.66, 1.39–1.98).

### 3.3. Secondary Outcome: Association between SAG and Readmission and MACEs

In this study, readmission rate and MACEs were regarded as the secondary outcome. Multivariate logistic regression indicated that increased SAG was associated with increased risk of MACEs in critically ill patients with CHF (tertile 3 versus tertile 1: OR, 95% CI: 1.76, 1.50–2.07). No obvious correlation between SAG and readmission rate was observed in critically ill patients with CHF (tertile 3 versus tertile 1: OR, 95% CI: 1.05, 0.88–1.24). The detailed data are shown in Supplementary Table 1.

### 3.4. Subgroup Analyses

Subgroup analyses were employed to assess the association between SAG and 30-day all-cause mortality. No significant interactions were found in most strata, as shown in Table 4, except for pneumonia, malignancy, respiratory failure, and mechanical ventilation. Among patients with CHF and high SAG, those with a comorbidity of pneumonia had a significantly lower 30-day mortality risk (HR, 95% CI: 1.44, 1.13–1.85 versus 3.27, 2.73–3.91). Similar trends also appeared in patients with a history of malignancy, respiratory failure, and mechanical ventilation (HR, 95% CI: 1.84, 1.10–3.07 versus 2.71, 2.33–3.15; 1.49, 1.22–1.83 versus 3.51, 2.85–4.32; 1.89, 1.55–2.30 versus 2.95, 2.38–3.65).

## 4. Discussion

CHF is a common critical illness in the field of cardiovascular disease, with characteristics of high prevalence, hospital admissions, readmissions, and even mortality [19]. According to the Framingham study, the 5-year survival rate of patients with CHF for men is only 25%, whereas that for women is 38%, which heavily threatens human health [20, 21]. Unfortunately, a lack of clinical objective indicators to predict the prognosis of patients with CHF exists at present. Various laboratory tests, such as blood gas analysis and biochemical testing, are often clinically used to determine the status of the acid-base balance of patients, but the relationship between these indicators and the prognosis of patients with CHF is rarely studied. Therefore, finding a simple and effective laboratory parameter to predict the prognosis of patients with CHF is particularly important and valuable for clinical treatment.

In this study, the possible prognostic value of SAG in patients with CHF was explored. Increased SAG was associated with increased risk of 30- and 90-day all-cause mortalities, and it remained as an independent predictor for all-cause mortality in critically ill patients with CHF after adjusting for age, gender, ethnicity, and another confounder. Besides, no significant interactions were found between SAG and most covariables for 30-day mortality, thus enhancing the stability and consistency of the results. To the best of the author's knowledge, this research was the first to clarify that high levels of SAG were associated with poor prognosis of critically ill patients with CHF.

As a routine examination that almost all admitted patients complete, SAG has the characteristics of simple calculation and easy acquisition, even without arterial puncture [14]. By retrospectively analyzing the electrolyte test results of 6868 hospitalized patients, Lolekha et al. [22] showed that there are SAG abnormalities in 40.5% of patients, including an increase in 37.6% of patients, and a decrease in 2.9%. Moreover, a series of studies in recent years further indicated that SAG could be used as a new prognosis biomarker to stratify patients at risk of deterioration, and patients could benefit from further treatment [5]. Sahu and his colleagues [23] reported that SAG acidosis was independently associated with in-hospital all-cause mortality in patients with AMI (OR, 95% CI: 4.2, 2.3–7.5), with longer average length of stay (5.1 versus 3.3 days). In critical ill patients with aortic aneurysm, Chen et al. [14] also demonstrated that SAG could effectively predict ICU mortality, and the risk of ICU death could increase by 38% for every increase of 1 mEq/L in SAG. Regrettably, the specific mechanism of SAG as a prognostic biomarker remains unclear.

The relationship between SAG and 30-day all-cause mortality in critically ill patients with CHF presented a U-shaped curve, which showed an elevated mortality risk at decreased and increased SAGs. In clinical practice, a decrease in SAG is usually rare, which may be related to the decrease in untested anions (such as hypoalbuminemia) or even laboratory error [24]. Albumin plays a remarkable role in various physiological processes, including maintaining colloidal osmotic pressure and microvascular integrity, ligand binding and material transport, antioxidant and antithrombotic effects, and enzyme activities [25]. Low level of blood albumin could promote and aggravate circulatory congestion and strengthen oxidative stress, inflammatory response, and susceptibility to infection, which could worsen the prognosis of patients with CHF [26]. In patients with acute heart failure, hypoalbuminemia has been proven to be associated with increased hospital mortality, and it served as an independent predictor of long-term mortality [27]. The increase in SAG is relatively more common, and previous studies have illustrated that the accumulation of serum lactate and ketone body accounted for 62% of the cause of the increase in SAG [28]. In CHF, the heart loses its ability to efficiently pump blood, which leads to decreased perfusion of tissues and hypoxia of cells [29]. Under anaerobic conditions, glucose undergoes glycolysis and eventually generates lactate, which may be primarily responsible for the increased SAG in patients with CHF [30]. In addition, sympathetic excitation in CHF contributes to the overproduction of lactate [31]. Under normal condition, the production and clearance of lactate are in balance, and this clearance is mainly responsible for the liver and kidney. Patients with CHF are often complicated by liver and kidney dysfunction, which further aggravates hyperlactataemia and the increase in SAG [32]. In the present study, the relationship between lactate and 30-day all-cause mortality in critically ill patients with CHF was also determined. Consistent with the results of previous studies [33], the increase in lactate was significantly correlated with poor prognosis (HR, 95% CI: 1.73, 1.53–1.95), which may be related to the damage and dysfunction of organs caused by tissue hypoperfusion and neurohormonal abnormalities. After adjusting for covariates including lactate, the anion gap was still associated with poor prognosis, thus showing the unique value of SAG as an independent predictor of the prognosis of critically ill patients with CHF.

This research has some limitations. First, it was a single-center study, whose subjects were relatively seriously ill. The results may not be applicable to all patients with CHF. Second, some unknown or even vital risk factors were not considered, although two multivariate models were used to control the influence of confounder on the outcome variables. Third, the SAG concentration of patients was only used when they entered the ICU to assess the relationship between them and all-cause mortality. It may be more valuable for the prognosis prediction if SAG can be dynamically monitored. Fourth, due to the lack of records of albumin, we did not perform albumin correction on the SAG, although some studies have shown that hypoalbuminemia could affect the SAG concentration [34]. Finally, the accuracy and efficiency of NT-proBNP and SAG in predicting the poor prognosis of critically ill patients with CHF were not compared, hence the possible exclusion bias.

## 5. Conclusion

SAG can effectively predict the 30- and 90-day all-cause mortalities of critically ill patients with CHF. It is expected to become a simple and effective marker for prognostic evaluation in these patients. Monitoring of SAG concentration could clinically help identify high-risk patients early and choose a more scientific treatment plan for patients.

## Figures and Tables

**Table 1:** Details of missing values.

Variables	The number of missing values	The percent of missing values	Variables	The number of missing values	The percent of missing values
SBP	39	0.5%	Glucose	48	0.6%
DBP	39	0.5%	Lactate	1993	26.8%
MBP	32	0.4%	NT-proBNP	6231	83.9%
RR	37	0.5%	PT	111	1.5%
HR	32	0.4%	APTT	137	1.8%
SpO_2_	34	0.5%	Sodium	1	<0.1%
Temperature	255	3.4%	Potassium	1	<0.1%
Weight	484	6.5%	Chloride	1	<0.1%
WBC	68	0.9%	Bicarbonate	2	<0.1%
Platelet	65	0.8%	Creatinine	2	<0.1%
Hemoglobin	88	1.1%	BUN	4	<0.1%

Note: SBP: systolic blood pressure; DBP: diastolic blood pressure; MBP: mean blood pressure; RR: respiratory rate; HR: heart rate; SpO_2_: percutaneous oxygen saturation; WBC: white blood cell; BUN: blood urea nitrogen; PT: prothrombin time; APTT: activated partial thromboplastin time; NT-proBNP: N-terminal probrain natriuretic peptide.

**Table 2:** The clinical characteristics of critically ill patients with CHF according to SAG levels.

Characteristics	Serum anion gap (mmol/L)			*P* value
	<13 (*n* = 2334)	≥13, <16 (*n* = 2519)	≥16 (*n* = 2573)	
Age (years)	72.2 ± 13.2	73.2 ± 13.2	72.5 ± 13.5	0.013
Gender, *n* (%)				0.278
Male	1280 (54.8)	1332 (52.9)	1359 (52.8)	
Female	1054 (45.2)	1187 (47.1)	1214 (47.2)	
Ethnicity, *n* (%)				0.002
White	1744 (74.7)	1881 (74.7)	1821 (70.8)	
Black	152 (6.5)	187 (7.4)	230 (8.9)	
Other	438 (18.8)	451 (17.9)	522 (20.3)	
SBP, mmHg	116.7 ± 15.2	117.6 ± 17.0	116.2 ± 17.8	0.004
DBP, mmHg	57.3 ± 9.4	58.0 ± 10.4	58.1 ± 10.9	0.013
MBP, mmHg	75.3 ± 9.7	76.1 ± 10.5	75.9 ± 11.1	0.038
HR, beats/minute	84.0 ± 14.2	84.0 ± 15.6	86.8 ± 16.7	<0.001
RR, beats/minute	18.7 ± 3.7	19.6 ± 3.9	20.2 ± 4.3	<0.001
Temperature, °C	36.8 ± 0.6	36.8 ± 0.6	36.7 ± 0.7	<0.001
SpO_2_, %	97.3 ± 1.9	96.9 ± 2.0	96.8 ± 2.4	<0.001
Weight, kg	81.9 ± 24.1	81.2 ± 24.3	81.3 ± 24.8	0.212
Scoring systems				
SOFA	4.5 ± 2.7	4.4 ± 2.8	5.7 ± 3.3	<0.001
SAPSII	37.1 ± 11.6	38.6 ± 12.6	44.2 ± 14.0	<0.001
Dialysis, *n* (%)	60 (2.6)	115 (4.6)	440 (17.1)	<0.001
Vasopressor, *n* (%)	558 (23.9)	498 (19.8)	666 (25.9)	<0.001
Ventilation, *n* (%)	624 (26.7)	784 (31.1)	1000 (38.9)	<0.001
ICU LOS, hours	116.7 ± 149.8	130.3 ± 164.5	147.4 ± 180.9	<0.001
30-day mortality, *n* (%)	254 (10.9)	399 (15.8)	665 (25.8)	<0.001
90-day mortality, *n* (%)	411 (17.6)	579 (23.0)	882 (34.3)	<0.001
Comorbidities, n (%)				
Liver diseases	97 (4.2)	89 (3.5)	119 (4.6)	0.144
Renal failure	325 (13.9)	552 (21.9)	813 (31.6)	<0.001
Atrial fibrillation	1053 (45.1)	1134 (45.0)	1116 (43.4)	0.377
Stroke	105 (4.5)	152 (6.0)	134 (5.2)	0.056
AMI	121 (5.2)	207 (8.2)	270 (10.5)	<0.001
VHD	178 (7.6)	248 (9.8)	264 (10.3)	0.003
PCD	130 (5.6)	140 (5.6)	139 (5.4)	0.959
HBP	278 (11.9)	467 (18.5)	673 (26.2)	<0.001
Diabetes	786 (33.7)	846 (33.6)	1021 (39.7)	<0.001
Pneumonia	440 (18.9)	581 (23.1)	640 (24.9)	<0.001
Respiratory failure	487 (20.9)	628 (24.9)	774 (30.1)	<0.001
Malignancy	94 (4.0)	145 (5.8)	118 (4.6)	0.015
Laboratory tests				
WBC (K/*μ*l)	11.4 ± 6.2	12.7 ± 27.8	13.8 ± 8.3	<0.001
Platelet (K/*μ*l)	196.2 ± 95.4	218.7 ± 98.2	240.1 ± 118.0	<0.001
Hemoglobin (g/dl)	10.2 ± 1.9	10.8 ± 2.0	10.9 ± 2.1	<0.001
Creatinine (mg/dl)	1.0 ± 0.6	1.4 ± 0.9	2.4 ± 2.0	<0.001
BUN (mg/dl)	23.2 ± 13.7	30.0 ± 19.0	45.1 ± 28.8	<0.001
Glucose (mg/dl)	129.0 ± 53.7	144.7 ± 60.9	171.3 ± 94.8	<0.001
Sodium (mmol/L)	138.8 ± 4.6	138.9 ± 4.6	138.2 ± 5.4	<0.001
Potassium (mmol/L)	4.1 ± 0.6	4.2 ± 0.7	4.4 ± 0.8	<0.001
Chloride (mmol/L)	106.2 ± 6.2	104.6 ± 5.9	102.7 ± 6.6	<0.001
Bicarbonate (mmol/L)	26.5 ± 4.9	24.8 ± 4.2	21.5 ± 4.7	<0.001
PT (second)	15.9 ± 7.1	16.7 ± 9.9	17.8 ± 12.0	<0.001
Laboratory tests				
APTT (second)	38.7 ± 23.8	38.8 ± 25.2	40.4 ± 26.6	0.002
Lactate, *n* (%)				<0.001
<1.7 mmol/L	1076 (46.1)	865 (34.3)	694 (27.0)	
≥1.7 mmol/L	634 (27.2)	858 (34.1)	1306 (50.8)	
No test	624 (26.7)	796 (31.6)	573 (22.3)	
NT-proBNP, *n* (%)				<0.001
<4983 pg/ml	192 (8.2)	219 (8.7)	186 (7.2)	
≥4983 pg/ml	118 (5.1)	200 (7.9)	280 (10.9)	
No test	2024 (86.7)	2100 (83.4)	2107 (81.9)	

Note: CHF: congestive heart failure; SAG: serum anion gap; SBP: systolic blood pressure; DBP: diastolic blood pressure; MBP: mean blood pressure; RR: respiratory rate; HR: heart rate; SpO_2_: percutaneous oxygen saturation; SOFA: stroke, and malignancy. Calculate the sequential organ failure assessment score; SAPSII: simplified acute physiology score II; ICU: intensive care unit; LOS: length of stay; AMI: acute myocardial infarction; VHD: valvular heart diseases; PCD: pulmonary circulation diseases; HBP: hypertension; WBC: white blood cell; BUN: blood urea nitrogen; PT: prothrombin time; APTT: activated partial thromboplastin time; NT-proBNP: N-terminal probrain natriuretic peptide.

**Table 3:** HRs (95% CIs) for all-cause mortality across groups of serum anion gap.

Variable	Crude		Model I		Model II	
	HR (95%CIs)	*P* value	HR (95%CIs)	*P* value	HR (95%CIs)	*P* value
30-day all-cause mortality						
Anion gap	1.10 (1.09, 1.11)	<0.0001	1.11 (1.10, 1.12)	<0.0001	1.05 (1.03, 1.07)	<0.0001
Anion gap (tertile)						
<13	1 (ref)		1 (ref)		1 (ref)	
≥13, <16	1.50 (1.28, 1.75)	<0.0001	1.47 (1.25, 1.72)	<0.0001	1.28 (1.09, 1.50)	0.0031
≥16	2.63 (2.27, 3.03)	<0.0001	2.62 (2.27, 3.03)	<0.0001	1.74 (1.46, 2.08)	<0.0001
*P* for trend	<0.0001		<0.0001		<0.0001	
Anion gap (quartile)						
<12	1 (ref)		1 (ref)		1 (ref)	
≥12, <14	1.41 (1.15, 1.73)	0.0009	1.39 (1.13, 1.70)	0.0016	1.22 (0.99, 1.49)	0.0621
≥14, <17	1.80 (1.50, 2.17)	<0.0001	1.75 (1.46, 2.11)	<0.0001	1.53 (1.26, 1.86)	<0.0001
≥17	3.16 (2.65, 3.78)	<0.0001	3.20 (2.68, 3.82)	<0.0001	1.97 (1.59, 2.45)	<0.0001
*P* for trend	<0.0001		<0.0001		<0.0001	
90-day all-cause mortality						
Anion gap	1.09 (1.07, 1.10)	<0.0001	1.09 (1.08, 1.10)	<0.0001	1.04 (1.02, 1.06)	<0.0001
Anion gap (tertile)						
<13	1 (ref)		1 (ref)		1 (ref)	
≥13, <16	1.36 (1.20, 1.54)	<0.0001	1.33 (1.17, 1.51)	<0.0001	1.17 (1.03, 1.34)	0.0170
≥16	2.22 (1.98, 2.50)	<0.0001	2.23 (1.98, 2.51)	<0.0001	1.53 (1.32, 1.77)	<0.0001
*P* for trend	<0.0001		<0.0001		<0.0001	
Anion gap (quartile)						
<12	1 (ref)		1 (ref)		1 (ref)	
≥12, <14	1.33 (1.14, 1.56)	0.0004	1.31 (1.12, 1.54)	0.0008	1.17 (0.99, 1.37)	0.0637
≥14, <17	1.57 (1.35, 1.82)	<0.0001	1.53 (1.32, 1.77)	<0.0001	1.35 (1.15, 1.57)	<0.0001
≥17	2.57 (2.23, 2.97)	<0.0001	2.62 (2.27, 3.02)	<0.0001	1.66 (1.39, 1.98)	<0.0001
*P* for trend	<0.0001		<0.0001		<0.0001	

Models were derived from Cox proportional hazards regression models. Crude model adjusted for none. Model I adjusted for age, gender, and ethnicity. Model II adjusted for age, gender, ethnicity, temperature, systolic blood pressure, diastolic blood pressure, respiratory rate, heart rate, percutaneous oxygen saturation, weight, atrial fibrillation, liver disease, valvular heart diseases, pulmonary circulation diseases, pneumonia, respiratory failure, diabetes, stroke, malignancy, lactate, prothrombin time, white blood cell, blood urea nitrogen, creatinine, potassium, bicarbonate, glucose, vasopressor, dialysis, mechanical ventilation, SOFA, SAPSII, and NT-proBNP. Note: HR: hazard ratio; CI: confidence interval; SOFA: stroke, and malignancy. Calculate the sequential organ failure assessment score; SAPSII: simplified acute physiology score II; NT-proBNP: N-terminal probrain natriuretic peptide.

**Table 4:** Subgroup analysis of the correlation between serum anion gap and 30-day all-cause mortality in critically ill patients with congestive heart failure.

	*N*	Serum anion gap (mmol/L)			*P* for interaction
		<13	≥13, <16	≥16	
Liver diseases					0.4454
No	7121	1.0 (ref)	1.45 (1.23, 1.70)	2.56 (2.20, 2.97)	
Yes	305	1.0 (ref)	1.98 (0.97, 4.06)	3.85 (2.04, 7.30)	
Renal failure					0.0908
No	5736	1.0 (ref)	1.56 (1.31, 1.85)	2.60 (2.21, 3.05)	
Yes	1690	1.0 (ref)	1.17 (0.79, 1.73)	2.74 (1.94, 3.88)	
Atrial fibrillation					0.1283
No	4123	1.0 (ref)	1.33 (1.07, 1.66)	2.25 (1.84, 2.76)	
Yes	3303	1.0 (ref)	1.62 (1.30, 2.03)	3.07 (2.50, 3.78)	
Stroke					0.6586
No	7035	1.0 (ref)	1.43 (1.21, 1.68)	2.63 (2.26, 3.06)	
Yes	391	1.0 (ref)	1.68 (1.01, 2.80)	2.61 (1.59, 4.27)	
AMI					0.4511
No	6828	1.0 (ref)	1.44 (1.23, 1.70)	2.58 (2.22, 2.99)	
Yes	598	1.0 (ref)	2.14 (1.02, 4.51)	3.90 (1.95, 7.83)	
VHD					0.0653
No	6736	1.0 (ref)	1.52 (1.28, 1.80)	2.78 (2.38, 3.24)	
Yes	690	1.0 (ref)	1.08 (0.69, 1.67)	1.63 (1.08, 2.46)	
PCD					0.0510
No	7017	1.0 (ref)	1.53 (1.30, 1.81)	2.77 (2.38, 3.22)	
Yes	409	1.0 (ref)	0.88 (0.51, 1.51)	1.43 (0.87, 2.34)	
HBP					0.2921
No	6008	1.0 (ref)	1.49 (1.26, 1.76)	2.59 (2.21, 3.03)	
Yes	1418	1.0 (ref)	1.61 (1.00, 2.58)	3.52 (2.29, 5.42)	
Diabetes					0.6109
No	4773	1.0 (ref)	1.41 (1.17, 1.69)	2.56 (2.20, 2.97)	
Yes	2653	1.0 (ref)	1.61 (1.19, 2.17)	2.76 (2.10, 3.62)	
Pneumonia					<0.0001
No	5765	1.0 (ref)	1.57 (1.29, 1.92)	3.27 (2.73, 3.91)	
Yes	1661	1.0 (ref)	1.16 (0.89, 1.50)	1.44 (1.13, 1.85)	
Respiratory failure					<0.0001
No	5537	1.0 (ref)	1.72 (1.37, 2.16)	3.51 (2.85, 4.32)	
Yes	1889	1.0 (ref)	1.06 (0.85, 1.32)	1.49 (1.22, 1.83)	
Malignancy					0.0320
No	7069	1.0 (ref)	1.42 (1.20, 1.68)	2.71 (2.33, 3.15)	
Yes	357	1.0 (ref)	1.69 (1.03, 2.77)	1.84 (1.10, 3.07)	
Dialysis					0.8370
No	6811	1.0 (ref)	1.45 (1.23, 1.70)	2.51 (2.16, 2.92)	
Yes	615	1.0 (ref)	1.48 (0.69, 3.19)	2.34 (1.18, 4.60)	
Vasopressor					0.0736
No	5704	1.0 (ref)	1.46 (1.21, 1.77)	2.33 (1.94, 2.80)	
Yes	1722	1.0 (ref)	1.62 (1.23, 2.13)	3.15 (2.48, 4.01)	
Ventilation					0.0088
No	5018	1.0 (ref)	1.57 (1.25, 1.98)	2.95 (2.38, 3.65)	
Yes	2408	1.0 (ref)	1.22 (0.99, 1.52)	1.89 (1.55, 2.30)	
SOFA					0.3416
<4	2785	1.0 (ref)	1.33 (1.00, 1.78)	2.01 (1.50, 2.69)	
≥4	4641	1.0 (ref)	1.56 (1.30, 1.89)	2.59 (2.19, 3.07)	
SAPSII					0.8125
<39	3701	1.0 (ref)	1.50 (1.13, 2.00)	2.17 (1.62, 2.91)	
≥39	3725	1.0 (ref)	1.36 (1.12, 1.64)	2.09 (1.77, 2.48)	

Note: AMI: acute myocardial infarction; VHD: valvular heart diseases; PCD: pulmonary circulation diseases; HBP: hypertension; SOFA: stroke, and malignancy. Calculate the sequential organ failure assessment score; SAPSII: simplified acute physiology score II.

## Data Availability

Publicly available datasets were analyzed in this study. This data can be extracted from Monitoring in Intensive Care Database III version 1.4 (MIMIC-III v.1.4) after passing on the required courses and obtaining the authorization.

## Supplementary Materials

Supplementary Materials Supplementary Table 1: ORs (95% CIs) for MACEs and readmission across groups of serum anion gap.
