# Supplementary material for: Serum Anion Gap Is Associated with All-Cause Mortality among Critically Ill Patients with Congestive Heart Failure
Source: Dis Markers. 2020 Nov 16;2020:8833637. doi: 10.1155/2020/8833637 (PMC7688352; doi:10.1155/2020/8833637)
Supplement: Supplementary Materials — Supplementary Table 1: ORs (95% CIs) for MACEs and readmission across groups of serum anion gap. [file 8833637.f1.docx]

Supplement Table 1. ORs (95% CIs) for MACEs and readmission across groups of serum anion gap.

| Variable | Crude | |  | | Model I | | |  | | Model II | | |  |
| --- | --- | --- | --- | --- | --- | --- | --- | --- | --- | --- | --- | --- | --- |
|  | OR (95%CIs) | P value | |  | | OR (95%CIs) | P value | |  | | OR (95%CIs) | P value | |
| MACEs |  |  | |  | |  |  | |  | |  |  | |
| Anion gap | 1.10 (1.09, 1.12) | <0.0001 | |  | | 1.11 (1.09, 1.12) | <0.0001 | |  | | 1.08 (1.06, 1.10) | <0.0001 | |
| Anion gap (tertile) |  |  | |  | |  |  | |  | |  |  | |
| < 13 | 1 (ref) |  | |  | | 1 (ref) |  | |  | | 1 (ref) |  | |
| ≥ 13, < 16 | 1.51 (1.34, 1.71) | <0.0001 | |  | | 1.50 (1.32, 1.70) | <0.0001 | |  | | 1.40 (1.22, 1.61) | <0.0001 | |
| ≥ 16 | 2.32 (2.06, 2.62) | <0.0001 | |  | | 2.35 (2.08, 2.66) | <0.0001 | |  | | 1.76 (1.50, 2.07) | <0.0001 | |
| P for trend | <0.0001 |  | |  | | <0.0001 |  | |  | | <0.0001 |  | |
| Readmission |  |  | |  | |  |  | |  | |  |  | |
| Anion gap | 1.01 (1.00, 1.03) | 0.0467 | |  | | 1.01 (1.00, 1.03) | 0.0662 | |  | | 1.01 (0.99, 1.03) | 0.4596 | |
| Anion gap (tertile) |  |  | |  | |  |  | |  | |  |  | |
| < 13 | 1 (ref) |  | |  | | 1 (ref) |  | |  | | 1 (ref) |  | |
| ≥ 13, < 16 | 1.06 (0.93, 1.21) | 0.3701 | |  | | 1.06 (0.93, 1.21) | 0.4030 | |  | | 1.04 (0.90, 1.20) | 0.5789 | |
| ≥ 16 | 1.11 (0.97, 1.27) | 0.1178 | |  | | 1.11 (0.97, 1.27) | 0.1236 | |  | | 1.05 (0.88, 1.24) | 0.5939 | |
| P for trend | 0.1187 |  | |  | | 0.1240 |  | |  | | 0.5836 |  | |

Models were derived from multivariate logistic regression. Crude model adjusted for none. Model I adjusted for age, gender, and ethnicity. Model II adjusted for age, gender, ethnicity, temperature, systolic blood pressure, diastolic blood pressure, respiratory rate, heart rate, percutaneous oxygen saturation, weight, atrial fibrillation, liver disease, valvular heart diseases, pulmonary circulation diseases, pneumonia, respiratory failure, diabetes, stroke, malignancy, lactate, prothrombin time, white blood cell, blood urea nitrogen, creatinine, potassium, bicarbonate, glucose, vasopressor, dialysis, mechanical ventilation, SOFA, SAPSII, and NT-proBNP.

Note: OR, odds ratio; CI, confidence interval; SOFA, stroke, and malignancy. Calculate the sequential organ failure assessment score; SAPSII, simplified acute physiology score II; NT-proBNP, N-terminal pro brain natriuretic peptide. MACEs, major adverse cardiac events.
